# Lens regeneration in axolotl: new evidence of developmental plasticity

**DOI:** 10.1186/1741-7007-10-103

**Published:** 2012-12-17

**Authors:** Rinako Suetsugu-Maki, Nobuyasu Maki, Kenta Nakamura, Saulius Sumanas, Jie Zhu, Katia Del Rio-Tsonis, Panagiotis A Tsonis

**Affiliations:** 1 Department of Biology and Center for Tissue Regeneration and Engineering, University of Dayton, 300 College Park, Dayton, OH 45469-2320, USA; 2 Current address: Institute of Protein Research, Osaka University, 3-2 Yamadaoka, Suita-Shi, Osaka 565-0871, Japan; 3 Current address: Faculty of Life and Environmental Sciences, University of Tsukuba, Tennoudai 1-1-1, Tsukuba, Ibaraki 305-8572 Japan; 4 Division of Developmental Biology, Cincinnati Children's Hospital Medical Center, 3333 Burnet Ave., Cincinnati, OH 45229, USA; 5 Department of Zoology, Miami University, 700 High Street, Oxford, OH 45056, USA

**Keywords:** Lens, Regeneration, Axolotl, Plasticity

## Abstract

**Background:**

Among vertebrates lens regeneration is most pronounced in newts, which have the ability to regenerate the entire lens throughout their lives. Regeneration occurs from the dorsal iris by transdifferentiation of the pigment epithelial cells. Interestingly, the ventral iris never contributes to regeneration. Frogs have limited lens regeneration capacity elicited from the cornea during pre-metamorphic stages. The axolotl is another salamander which, like the newt, regenerates its limbs or its tail with the spinal cord, but up until now all reports have shown that it does not regenerate the lens.

**Results:**

Here we present a detailed analysis during different stages of axolotl development, and we show that despite previous beliefs the axolotl does regenerate the lens, however, only during a limited time after hatching. We have found that starting at stage 44 (forelimb bud stage) lens regeneration is possible for nearly two weeks. Regeneration occurs from the iris but, in contrast to the newt, regeneration can be elicited from either the dorsal or the ventral iris and, occasionally, even from both in the same eye. Similar studies in the zebra fish concluded that lens regeneration is not possible.

**Conclusions:**

Regeneration of the lens is possible in the axolotl, but differs from both frogs and newts. Thus the axolotl iris provides a novel and more plastic strategy for lens regeneration.

## Background

Salamanders, especially newts, are capable of regenerating tissues, organs and even body parts, such as limbs and tails throughout their adult lives [1,2]. Among all tissues that can be regenerated, the lens is a special case. In contrast to removal of a part of limb or tail (total removal disallows regeneration), the lens can be removed in its entirety. It has also been shown that newts can regenerate the lens very faithfully no matter how many repeated lentectomies are performed or the age of the animal [3]. Notably, the lens regenerates by another tissue, the pigment epithelial cells of the iris by the process of transdifferentiation. There is, however, a restriction: The lens can only be regenerated from the dorsal iris. Even though the ventral iris can be induced experimentally to regenerate a lens, normally it never contributes to the process [4]. This restriction can be very instructive when it comes to investigating the mechanisms underlying the process.

The only other vertebrates that have been shown to regenerate the lens are frogs and some fish. In frogs, the process has been studied well and it has been established that regeneration occurs only in pre-metamorphic stages and that the lens is derived from the cornea by transdifferentiation [5]. After metamorphosis, the ability for lens regeneration ceases. Another salamander, the neonate axolotl, is also capable of regenerating limbs and tails as is the newt. However, in a paper by Stone in 1967 it was concluded that this species is not able to regenerate the lens, even though no staging details were provided [6]. We have decided to revisit this issue and have undertaken a detailed study starting with larvae at stage 44. At this stage the eye tissues including the lens have been well differentiated, while other body parts (such as limbs) are beginning to form [7]. We find that starting at this stage axolotls, similarly to newts, can regenerate a perfect lens from the iris and that this ability persists for about two to three weeks beyond that stage. After that, the ability for lens regeneration is lost. Surprisingly, however, we find that the lens can be regenerated by either the dorsal or the ventral iris. In some cases regeneration occurred from both irises in the same eye. A similar series of experiments employing zebrafish failed to show any evidence that this species can regenerate their lens underscoring the importance of urodeles in the study of lens regeneration.

## Methods

### Lentectomy

Axolotl larvae (st35 and st43, pre-hatched) were supplied from the Ambystoma Genetic Stock Center (Department of Biology, Univ. of Kentucky, KY, USA) and kept at 27°C. The hatched larvae were fed brine shrimp. Larvae were anesthetized in 0.1% ethyl 3-aminobenzoate (#E10521; Sigma, St. Louis, MO, USA) at different stages. Using a sharp-edged blade, an incision was made in the cornea, and then the lens was removed in its entirety. At different time intervals after lentectomy, animals were fixed in methanol acetic acid solution (methanol: acetic acid = 3:1) at 4°C overnight and processed for paraffin embedding. Similar series were also performed using zebrafish embryos (wild-type Ekkwill strain) at different stages. Animal care adhered to the guide lines of the Institutional Animal Care and Use Committee (IACUC), University of Dayton (axolotl) and of the IACUC, Cincinnati Children's Hospital Medical Center (zebrafish).

### Hematoxylin and Eosin staining and immunohistochemistry

Paraffin sections of 15 μm were deparaffinized and used for H & E staining and immunohistochemistry. For H & E staining, hematoxylin (#26754-01; Electron Microscope Sciences, Hatfield, PA, USA) and eosin (#26762-01; Electron Microscope Sciences) were used. To examine cell proliferation in the iris after lentectomy, 5-bromo-2'- deoxyuridine (BrdU; #B5002; SIGMA, 75 ug/g body weight) was injected into larvae 3 hours before fixation. Sections from BrdU injected larvae were treated with 1N HCl for 5 minutes at room temperature, blocked in TNB buffer (0.1 M Tris-HCl, pH7.5; 0.15 M NaCl; and 0.5% blocking reagent) supplied in the TSA kit (Perkin Elmer, Waltham, MA, USA) and incubated with mouse anti-BrdU antibody (1/100 dilution, #MAB3510; Millipore, Billerica, MA, USA) overnight at 4°C, and subsequently washed and incubated with Alexa 488 conjugated anti-immunoglobulin G (IgG) (1/100 dilution, Invitrogen, Grand Island, NY, USA) for 90 minutes at room temperature. To monitor lens regeneration, sections were incubated with anti γ-crystallin rabbit antibody (1/300 dilution, source bovine crystallin) and then detected with an anti-rabbit Cy3 conjugated antibody (1/100 dilution, Millipore). Immunohistochemistry images were taken using a BX51 microscope (Olympus, Tokyo, Japan) with a CCD camera (Cool SNAP cf2; Photometrics, Tucson, AZ, USA) and imaging software (Metamorph, Molecular Devices, Eugene, OR, USA), or by confocal imaging (Olympus FV500 confocal microscope).

## Results and discussion

**Figure 1:**
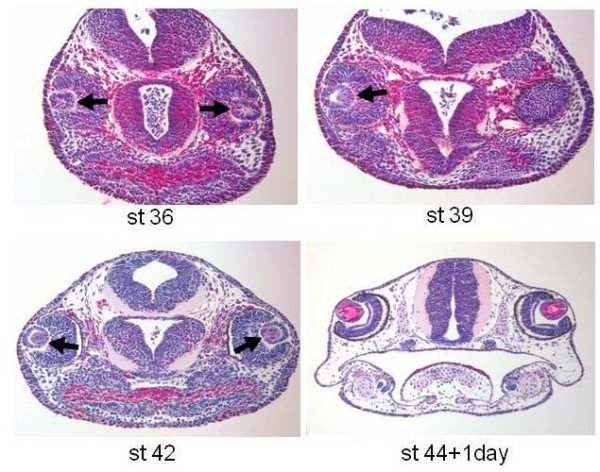
**Histological sections through the head of developing axolotls at different stages, to show the degree of differentiation of the eye and especially the lens**. At stage 36, there is only a lens vesicle (arrows) with no clear differentiation of lens fibers yet. At stage 39, cells at the posterior part of the lens vesicle start elongating (arrow) and differentiating to lens. This is the beginning of lens fibers differentiation. At stage 42, differentiation of lens fibers covering most of the lens is evident (red color, arrow). At stage 44 +1 the lens is fully differentiated with a lens epithelium in the anterior region and differentiated lens fibers at the posterior region. H & E staining.

**Figure 2:**
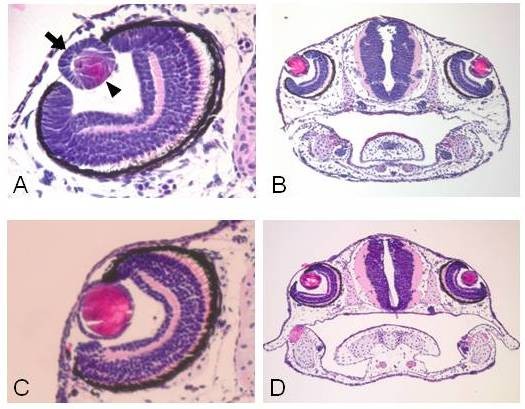
**Examples of lens regeneration in axolotl larvae**. **A**: A regenerating lens 2 days after lentectomy (lentectomy was performed at stage 44 + 2 days). Note that the lens is attached to the iris and shows the characteristic structure of the lens epithelium at the anterior (arrow) and differentiated lens fibers at the posterior region (arrowhead). **B**: A section through the head of an un-operated animal at the same stage as in A (in this case stage 44 + 4 days) to compare the degree of lens differentiation. **C**: A regenerated lens 7 days after lentectomy (lentectomy was performed at stage 44 + 3 days). Note an almost complete differentiation of lens. **D**: A section through the head of an un-operated animal at the same stage as in C (in this case stage 44 + 10 days) to compare the degree of lens differentiation. H & E staining.

Based on the embryonic stages of axolotl outlined by Armstrong and Malacinski [7], we histologically evaluated lens development in embryos as early as stage 36. Our evaluation showed that by stage 44, the lenses had developed well with full differentiation of the globe having a clear lens epithelium and lens fibers (Figure 1). Since this is the stage at which hatching occurs (and no further staging is available), we decided to remove the lens at different time intervals after stage 44 starting with day 1 to day 27. Each group was examined at different times after lentectomy to evaluate if animals were able to regenerate their lenses. We found that lens regeneration was possible within a particular time window after lens removal in animals up to 14 days after hatching (stage 4; Table 1, Figure 2). After that window of time, axolotls were found incompetent of regenerating their lenses. The regeneration process was very fast. Within one to two days after lentectomy, a well differentiated lens was present. The frequency of lens regeneration was highest when lentectomy was performed 3 or 7 days after stage 44; there were 10/16 (62.5%) and 8/10 (80%) regenerated lenses, respectively. We believe that the failed cases were most likely the result of trauma to the eye due to lentectomy. The eye of the axolotl at these stages is very small and no matter how carefully lentectomy is performed some injury is unavoidable. When we examined later stages, such as 27 days past stage 44, axolotls were no longer able to regenerate the lens. In Table 2 in parentheses we include staging beyond stage 44, according to limb development [8].

**Table 1:** Lens regeneration in axolotl evaluated at different stages starting with 1 day post-hatch after stage 44.

Stage at lentectomy	Regenerated lenses
44+1day (44-45)	4/8
44+2days (46-48)	4/8
44+3days (48-49)	10/16
44+7days (49-50)	8/10
44+10days (50-51)	4/16
44+13days (51-52)	2/4
44+27days (>54)	0/10

In parentheses equivalent stages as described by Nye *et al. *according to forelimb and hindlimb development [8].

**Table 2:** Lens regeneration in zebrafish at different stages of development.

Stage	Regenerated lenses at Day 4 post-lentectomy
Prim 22 (35 hpf)	0/9
High or Long Pec (42-48 hpf)	0/5
Long Pec + FGF2 (48 hpf)	0/17
Protruding mouth (72 hpf)	0/2

Hatching is around 42 hpf.

**Figure 3:**
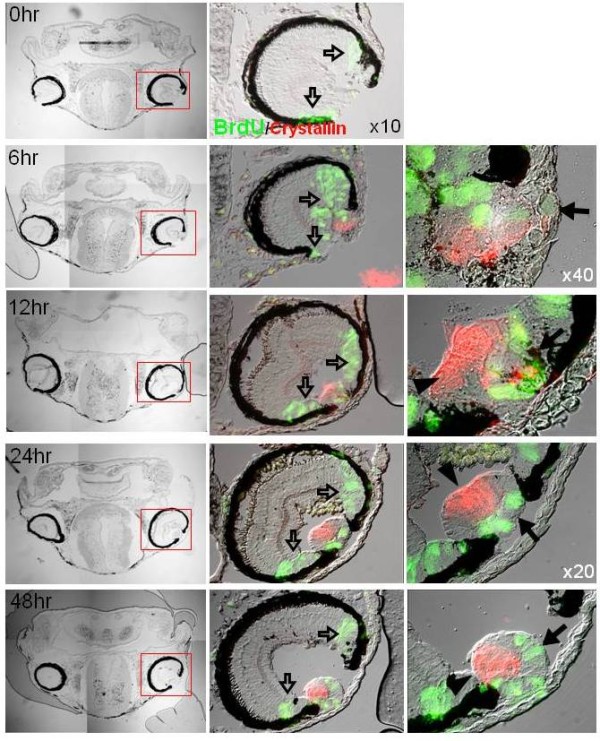
**A detailed analysis of lens regeneration, showing lens proliferation and crystalline synthesis**. All lentectomies were performed at stage 44 + 8 days. The first column shows bright field sections through the heads and the eyes. The second column shows bright field sections with BrdU staining (green) and γ-crystallin staining (red) X10. The third column shows higher magnification images to depict better the regenerating lenses. The different rows represent samples at different time points after lentectomy. Note that the regenerating lens comes from the dorsal iris (the case at 6 hours) or the ventral iris (case at 48 hours). The regenerating lens is obvious at 6 hours post-lentectomy and starts organizing well with proliferating lens epithelium at the anterior (arrows) and differentiating lens fibers at the posterior (arrowheads) as soon as 12 hours post-lentectomy. The proliferating cells in the ciliary margin (open arrows) are retina stem/progenitor cells that contribute to the growing retina.

**Figure 4:**
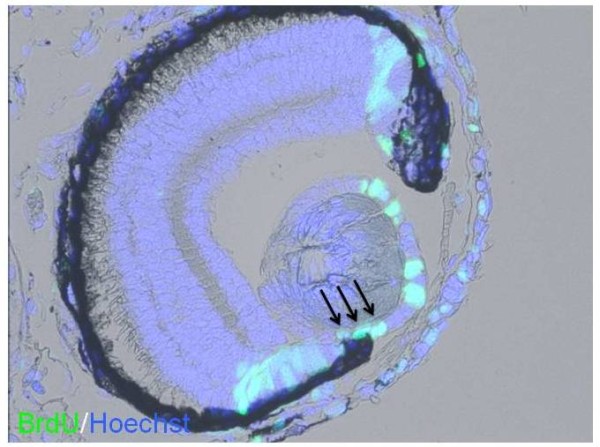
**A different case of regenerating lens 24 hours after lentectomy (lentectomy was performed at stage 44 + 8 days**. The regenerated lens is clearly attached to the iris (ventral) whose cells at the tip are actively dividing (arrows). As expected many other cells of the anterior lens epithelium are dividing as well. X40.

**Figure 5:**
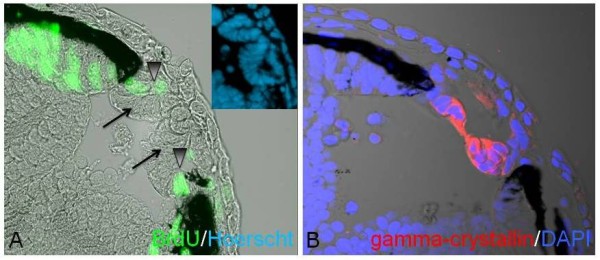
**Two cases of lenses regenerated from both dorsal and ventral iris seen at 6 hours after lentectomy (lentectomy was performed at stage 44 + 8 days)**. **A**: note two early lens vesicles (arrows) with proliferating cells (arrowheads) at the tip of both irises. In the insert the same vesicles are shown stained with Hoerstch. **B**: Another case with two regenerating lenses. Blue is 4',6-diamidino-2-phenylindole (DAPI) and red is γ-crystallin. X40.

Histological sections at day 1 after lentectomy hinted that the lens regenerates from the iris; however, it could be formed from either the dorsal or the ventral iris. Prompted by these findings we decided to analyze lens regeneration in more detail. For this, we removed the lens at stage 44 + 8 days and analyzed the process of regeneration 3, 6, 12, 24 and 48 hours post-lentectomy. Three hours before collection, animals were injected with BrdU to examine cell proliferation. The collected animals were embedded, sectioned and incubated with BrdU antibody as well as with γ-crystallin antibody that marks lens fibers. The histological series are presented in Figure 3. In the top panel, sections at 0 hour, just after removing the lens, are shown. BrdU staining was only observed at the ciliary margin, where proliferating retinal stem/progenitor cells contribute to the growing retina. As expected, γ-crystallin expression is absent. At 3 hours post-lentectomy no crystallin synthesis was documented (not shown). At 6 hours post-lentectomy a lens vesicle positive for γ-crystallin is present. The vesicle becomes more organized and polarized at 12, 24 and 48 hours with an anterior region having proliferating lens epithelial cells and a posterior part with differentiating lens fibers. This represents the normal process of lens development as well. Interestingly, the lens was elicited from the dorsal iris (9/14, 64.3%) or the ventral iris (3/14, 21.5%). Most surprisingly, however, is that in 2/14 (14.2%) cases lenses were regenerated from both the dorsal and the ventral iris. The regenerating lens at 6 hours originated from the dorsal iris, while the regenerating lens at 48 hours originated from the opposite, the ventral, iris (Figure 3). In a different eye, a regenerating lens 24 hours after lentectomy was produced from the ventral iris (Figure 4). The lens was attached to the tip of the iris, which was also positive for BrdU. The lens epithelium was also positive as expected. The two cases of the double lenses can be seen in Figure 5. In Figure 5A two lens vesicles derived from both dorsal and ventral iris are depicted and in Figure 5B two crystallin-positive lenses can be observed.

Our results, thus, convincingly show that lens regeneration is possible in the axolotl larvae. In this respect, there are differences and similarities when axolotl lens regeneration is compared with the same process in frogs and in newts. Similarly to frogs, regeneration is possible during a limited time window, but in contrast, the iris is the source of regeneration in the axolotl and not the cornea. Similarly to newts, regeneration takes place from the iris pigment epithelium, but in contrast to the newt, it is not restricted to only the dorsal iris, and this capacity is not present at later stages or as an adult.

It is interesting to also note here that the competent stages for lens regeneration coincide with early development of forelimbs and hindlimbs. Stage 44 is morphologically marked by the development of the forelimb bud [7,8]. The capacity for regeneration is terminated around stage 54 which is marked by the beginning of hindlimb bud differenrtiation. While a direct correlation between the two events cannot be made at present, one idea could be that crucial factors for both events act during that time window.

**Figure 6:**
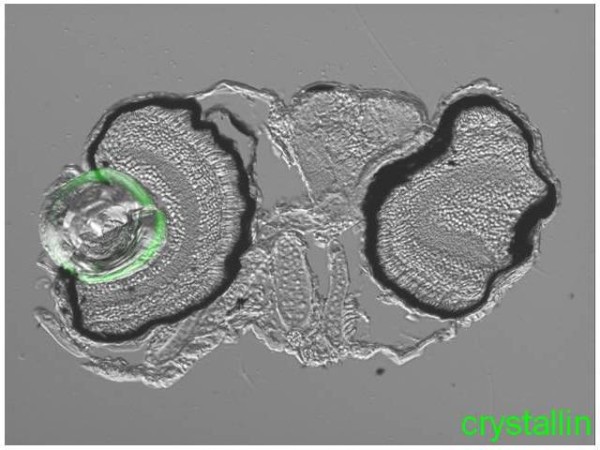
**An example of lack of lens regeneration in zebrafish embryo**. Lentectomy was performed at Long Pec Stage (48 hpf). Only the lens from the right eye was removed. The lens on the left eye was not touched. There is no sign of lens regeneration or crystallin synthesis (green) in the lentectomized eye when the animal was examined three days after lentectomy. Note that there was no severe trauma in the lentectomized eye.

Prompted by the axolotl results, we also examined lens regeneration in zebrafish. Lens formation in zebrafish with well differentiated anterior lens epithelium and posterior lens fibers occurs within 28 hours post fertilization [9]. We removed lenses at different stages starting at prim 22 (35 hpf), high pec (42 hpf) or long pec (48 hpf) and protruding mouth (72 hpf). Hatching begins at high pec stage. In none of the cases was a regenerated lens obtained (Table 2, Figure 6). Given the role of fibroblast growth factor (FGF) in zebrafish fin regeneration [10] and in newt lens regeneration [11,12], we also added this growth factor to zebrafish embryos or to axolotls at non-permissive stages post-lentectomy. FGF failed to induce any regeneration (not shown).

Taken together our results underscore the importance of newts in the study of lens regeneration. However, the fact that there is a time window in which the axolotl can actually regenerate the lens provides an excellent comparative addition to the study of lens regeneration. Recently, the transcriptome of newt lens regeneration has been completed (unpublished) as well as similar studies during axolotl limb regeneration [13]. Thus, this technology could be of great value to regeneration studies. It will be of great interest to compare, in the future, the transcriptome of lens regeneration in axolotl (permissive and non-permissive stages) with the newt transcriptome of the dorsal and ventral iris. Such analysis can pinpoint crucial regulation underlying induction of lens regeneration.

## Conclusions

In this study, we have shown that lens regeneration is possible in the axolotl for a limited period of time. Lens can be regenerated between stage 44 (hatching) and stage 52 (2 weeks post hatching). Our results show that contrary to the newt that regenerates the lens only from the dorsal iris, the lens can be regenerated from either the dorsal or the ventral iris or even from both sites. Thus, the axolotl lens regeneration provides evidence for a new developmental plasticity.

## Abbreviations

BrdU: 5-bromo-2'-deoxyuridine; FGF: fibroblast growth factor; H & E: hematoxylin and eosin.

## Competing interests

The authors declare that they have no competing interests.

## Authors' contributions

RS-M and NM designed the research, performed the experiments and analyzed data. KN, SS, JZ and KDRT performed experiments and analyzed data. PAT designed the research, analyzed data and wrote the paper. All authors have read and approved the manuscript for publication.
